# SKELETAL MUSCLE MITOCHONDRIAL PHYSIOLOGY IN CHILDREN WITH CEREBRAL PALSY: CONSIDERATIONS FOR HEALTHY AGING

**DOI:** 10.1093/geroni/igac059.516

**Published:** 2022-12-20

**Authors:** Sudarshan Dayanidhi

**Affiliations:** Shriley Ryan AbilityLab/Northwestern University, Chicago, Illinois, United States

## Abstract

During healthy aging, there is an overall decline in mitochondrial activity and abundance, increase in mitochondrial DNA mutations, increase in oxidative stress, and reduction in overall muscular capacity. Individuals with cerebral palsy (CP) have significantly increased energetics of movement, reduced endurance capacity, and increased perceived effort. We will cover the results of recent work in muscles in ambulatory children with CP that show a marked reduction in mitochondrial function. Muscles show that mitochondrial protein content and DNA copy number are lower, suggesting a reduction in mitochondrial abundance, along with a reduction in markers for mitochondrial biogenesis. Gene expression networks are reduced for glycolytic and mitochondrial pathways and share similarities with gene networks with aging and chronic inactivity. Given the importance of mitochondria for energy production and changes with aging, ongoing efforts are needed to assess changes in mitochondria across the lifespan in people with CP.

